# The Quest for a Truly Universal Influenza Vaccine

**DOI:** 10.3389/fcimb.2019.00344

**Published:** 2019-10-10

**Authors:** Yo Han Jang, Baik Lin Seong

**Affiliations:** ^1^Molecular Medicine Laboratory, Department of Biotechnology, College of Life Science and Biotechnology, Yonsei University, Seoul, South Korea; ^2^Vaccine Translational Research Center, Yonsei University, Seoul, South Korea

**Keywords:** influenza virus, universal influenza vaccine, cross-protection, HA stalk, M2e, T cell, live attenuated influenza vaccine

## Abstract

There is an unmet public health need for a universal influenza vaccine (UIV) to provide broad and durable protection from influenza virus infections. The identification of broadly protective antibodies and cross-reactive T cells directed to influenza viral targets present a promising prospect for the development of a UIV. Multiple targets for cross-protection have been identified in the stalk and head of hemagglutinin (HA) to develop a UIV. Recently, neuraminidase (NA) has received significant attention as a critical component for increasing the breadth of protection. The HA stalk-based approaches have shown promising results of broader protection in animal studies, and their feasibility in humans are being evaluated in clinical trials. Mucosal immune responses and cross-reactive T cell immunity across influenza A and B viruses intrinsic to live attenuated influenza vaccine (LAIV) have emerged as essential features to be incorporated into a UIV. Complementing the weakness of the stand-alone approaches, prime-boost vaccination combining HA stalk, and LAIV is under clinical evaluation, with the aim to increase the efficacy and broaden the spectrum of protection. Preexisting immunity in humans established by prior exposure to influenza viruses may affect the hierarchy and magnitude of immune responses elicited by an influenza vaccine, limiting the interpretation of preclinical data based on naive animals, necessitating human challenge studies. A consensus is yet to be achieved on the spectrum of protection, efficacy, target population, and duration of protection to define a “universal” vaccine. This review discusses the recent advancements in the development of UIVs, rationales behind cross-protection and vaccine designs, and challenges faced in obtaining balanced protection potency, a wide spectrum of protection, and safety relevant to UIVs.

## Introduction

**Figure 1:**
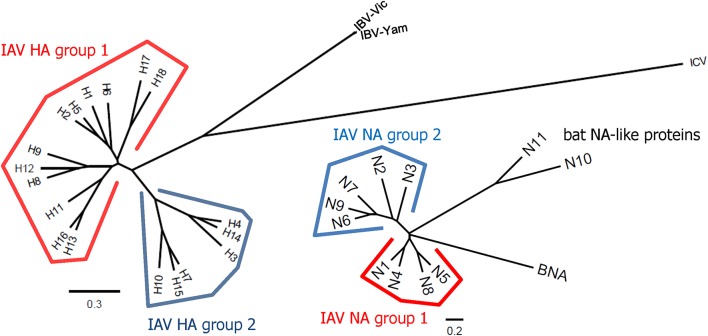
Phylogenetic trees representing HA and NA diversity among influenza viruses. The 18 subtypes of HAs of IAV are divided into two phylogenetic groups according to their amino acid sequences similarities. The HAs of IBVs are divided into Victoria-like and Yamagata-like lineages but they are closer to each other than any of two different subtypes of IAVs. The HAs of ICVs are antigenically distant from those of IAVs and IBVs. The NAs of IAVs also show high levels of antigenic variability and are divided into two groups. Phylogenetic trees were constructed based on amino acid sequence comparisons among influenza viruses. Multiple alignments were carried out using the representative sequence of each HA or NA subtype or lineage. The phylogenetic trees were constructed by the ClustalW algorithm using neighbor joining (N-J) method and visualized by FigTree v1.4.4. The scale bars represent amino acid change (%).

Influenza viruses present a high level of antigenic diversity and variability due to their segmented RNA genome. These viruses are classified into four major types, A, B, C, and D, based on their nucleoprotein (NP) and matrix (M) genes. Human infecting type A and B viruses are further classified into multiple subtypes or lineages, respectively, depending on the antigenicity of viral surface proteins, hemagglutinin (HA), and neuraminidase (NA) genes (Paules and Subbarao, 2017). Influenza A and B viruses co-circulate in every season and, thus, c represents the primary targets of seasonal influenza vaccines (Sridhar et al., 2015). In addition to seasonal epidemics, influenza viruses have caused pandemics at the intervals of ~10–40 years since the 1918 Spanish flu H1N1, the 2009 pandemic H1N1 being the last outbreak (Saunders-Hastings and Krewski, 2016). While vaccination remains the most cost-effective measure to prevent influenza virus infections, antigenic drift in the surface antigens allows these viruses to escape antibody-mediated neutralization (Kim et al., 2018a). In addition, the sudden occurrence of pandemics is often accompanied by zoonotic spillover of the surface genes into the human-infecting viruses, rendering preexisting vaccines ineffective to newly emerging viruses. The variation caused by genetic drift and shift is unpredictable, posing a serious challenge to the management of influenza outbreak. Based on the amino acid sequences of HAs, influenza A viruses (IAVs) are divided into two phylogenetic groups. The IAV HA group 1 viruses include H1, H2, H5, H6, H8, H9, H11, H12, H13, H16, H17, and H18, while the group 2 viruses comprise H3, H4, H7, H10, H14, H15 (Figure 1). The NAs of IAVs are also antigenically diverse, presenting two distinct groups. Influenza B viruses (IBVs) are not divided into subtypes but circulate as two distinct Yamagata-like and Victoria-like lineages. Influenza C viruses (ICVs) generally cause a mild respiratory disease in humans and do not cause epidemics (Dykes et al., 1980). Contemporary influenza epidemics are caused by the H1N1 and H3N2 of the IAVs and one or two lineages of the IBVs, dictating trivalent (TIV) or quadrivalent influenza vaccine (QIV) containing two IAV antigens and one or two IBV antigens, respectively (Ambrose and Levin, 2012).

**Figure 2:**
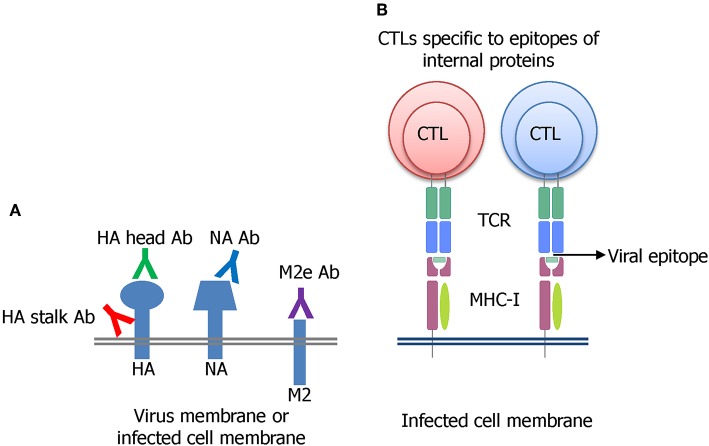
Major principles of developing a UIV. **(A)** Induction of broadly protective antibodies specific to conserved regions in the HA stalk or head, NA, or M2e has been extensively investigated. The antibodies bind to viral surface proteins either on virion or expressed on virus-infected cell membrane. **(B)** Cytotoxic T lymphocyte (CTL) recognizing the conserved epitopes of influenza internal proteins such as NP, M1, or PB1 has been shown to be critical for broad protection through the clearance of the virus-infected cells.

Many strategies have been undertaken on the pursuit of developing a universal influenza vaccine (UIV) (Paules et al., 2017). The induction of cross-protective immune responses directed toward conserved B cell or T cell epitopes is a major principle underlying broad protection (Figure 2). The direct binding of antibodies to the viral surface proteins interferes with their functions and results in virus neutralization before cell entry (Figure 2A). Alternatively, the antibodies may bind to viral antigens displayed on the surface of virus-infected cells and mediate effector functions to remove the infected cells. A cytotoxic T lymphocyte (CTL) can kill the virus-infected cells in an independent manner (Figure 2B). Several conserved viral antigens such as M2 extracellular domain (M2e), HA stalk and receptor binding site, NA, and T cell epitopes in the internal proteins such as polymerase basic protein 1 (PB1), NP, and M1 were defined as targets for eliciting cross-reactive immune response. A variety of vaccine platforms were tested for effective exposure of those antigens to the immune system (Wang et al., 2018a; Estrada and Schultz-Cherry, 2019). While these vaccines showed broader cross-protection than the classical inactivated influenza vaccines (IIVs), their protection potency was weak. They provided partial protection against the antigenically distant viruses, and the protection breadth is usually limited to the same group (subtype-specific or HA group-specific) and not effective in another HA group. Thus, there exists a considerable gap between the current status, and the ultimate goal, of a truly universal vaccine.

Some studies have discovered rare antibodies specific to the conserved HA stalk in animals and humans with prior exposure to influenza viruses. Some of these antibodies show extremely broad specificity, encompassing both HA groups of IAVs (Ekiert et al., 2011) or even both IAVs and IBVs (Dreyfus et al., 2012), representing an optimistic prospect of developing a UIV. However, no studies so far have reported a successful vaccine approach that induces such antibodies to a protective level, reflecting considerable difficulty in selectively inducting particular antibodies in the context of vaccination. While antibody-dependent effector mechanisms and T cell immunity have emerged as potential correlates of broad protection against influenza infections (Krammer and Palese, 2015), the exact molecular mechanisms of their protective action are not completely understood, and immunopathology by T cell responses still remains a challenge (Peiris et al., 2010; Duan and Thomas, 2016; Erbelding et al., 2018). Moreover, understanding how preexisting immunity (immune imprinting or antigenic sin-like phenomenon) shaped by prior exposure to influenza viruses affects the magnitude, hierarchy, and sustainability of antibody response to vaccination is suggested as critical for designing a UIV in humans (Henry et al., 2018). The involvement of multiple factors in eliciting broad protection and the influence of pre-existing immunity on the subsequent vaccination pose a dilemma on establishing the correlate of cross-protection against heterologous or hetero-subtypic viruses, representing a critical obstacle for the licensure of a UIV by the regulatory authorities.

This review updates the recent advances in UIV development and focuses on the critical issues to be addressed in designing a ‘truly’ UIV. Alternative vaccine antigens and vaccine strategies for durable and broader protection are also discussed in detail. This review is meant to help the readers to acquire general information on the cutting edge of UIV researches and to gain wide perspectives on rational design of a truly UIV with improved potency, breadth, and safety.

## Criteria for a UIV

### Definition of Universal Protection

**Figure 3:**
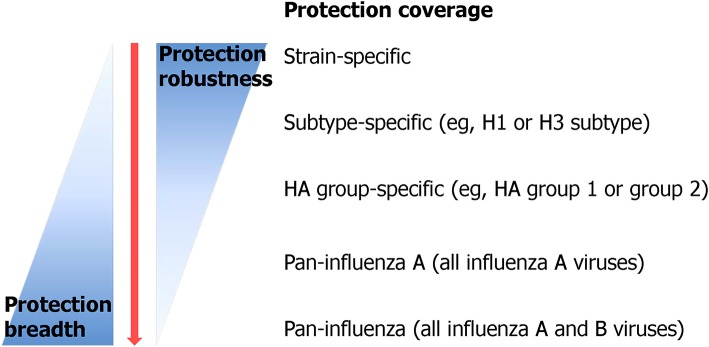
Protection breadth of influenza vaccines ranging from strain-specific protection to pan-influenza universal protection. Currently licensed seasonal influenza vaccine provides only strain-specific protection against well-matched strains. Recently, many efforts have been put in order to improve the protection breadth of a vaccine from subtype-specific protection to, ideally, pan-influenza universal protection [adopted from Erbelding et al. (2018)].

Although there is a clear consensus about the urgent need for a vaccine that provides durable and broad protection against multiple strains of influenza virus, the definition of the “universality” of UIV is still debated (Krammer et al., 2018b). Recently, the National Institute of Allergy and Infectious Diseases (NIAID) held a meeting to identify and develop the criteria to define a UIV. The participants from multiple disciplines agreed that a reasonable UIV should be at least 75% effective against symptomatic influenza virus infection caused by group 1 and group 2 IAVs, with the protection lasting over 1 year for all age groups (Paules et al., 2017; Erbelding et al., 2018) (Table 1). Similar criteria have also been suggested as preferred product characteristics (PPC) and target product profiles (TPP), describing the desired characteristics of a UIV, by the World Health Organization (WHO) and the Bill and Melinda Gates Foundation (BMGF), respectively (Table 1). Despite the priority given to IAVs in WHO PPC (Table 1), the IBVs represent a “low hanging fruit” because of their low antigenic diversity and the lack of animal reservoir, presenting credence to potential eradication from humans (Tan et al., 2018). The proposed consensus to define universal protection offers a valuable guideline to develop an effective and safe UIV. Ideally, the spectrum of protection may covers both IAVs and IBVs, considering the existence of extremely broadly protective antibodies and T cell epitopes across IAVs and IBVs from animals and humans (Corti et al., 2011; Koutsakos et al., 2019). However, as the antigenic difference between a vaccine and a target virus becomes larger, the number of conserved B cell or T cell epitopes between the two strains decreases. The limited availability of target epitopes may result in compromised protection robustness due to (1) decreased clonal diversity of cross-reactive antibodies and T cells, or (2) occurrence of escape mutants by even small amount of genetic mutations in the epitopes. Therefore, a balanced breadth and robustness of cross-protection should be considered when developing a reliable UIV (Figure 3).

**Table 1:** Consensus criteria for a UIV.

	**NIAID**	**WHO**	**BMGF**	**Consensus**
Breadth	All influenza A viruses (influenza B protection would be the second target)	All influenza A viruses	All influenza A and B viruses	All influenza A viruses
Efficacy	At least 75% effective against symptomatic influenza infection	Better than that of current seasonal influenza vaccine	At least 70% effective against symptomatic influenza infection	At least 70% effective
Target population	All age groups	>6 weeks, no upper age limit including high risk groups	>6 weeks, no upper age limit including high risk groups	>6 weeks, no upper age limit
Duration of protection	At least 1 year	At least 5 years	3–5 years	At least 1 year

*NIAID, National Institute of Allergy and Infectious Diseases (https://www.niaid.nih.gov/diseases-conditions/universal-influenza-vaccine-research); BMGF, Bill and Melinda Gates Foundation (https://gcgh.grandchallenges.org/challenges). WHO, World Health Organization (https://apps.who.int/iris/bitstream/handle/10665/258767/9789241512466-eng.pdf; https://jsessionid=37D9E056C3EA58A90EE237432AA7D65F?sequence=1)*.

The NIAID and the researchers have proposed three research areas to address the knowledge gaps in developing a UIV; (1) understanding of influenza transmission, natural history, and viral pathogenesis, (2) characterization of correlates of protection, and (3) rational design of a UIV to improve potency and breadth of protection (Erbelding et al., 2018). In particular, the correlates of protection elicited by a UIV may vary considerably in quantity and quality, depending on the vaccine type used and the target influenza virus tested. For instance, while the protection potency of HA stalk-based vaccine may easily be evaluated by measuring the neutralizing activity or indirect effector mechanisms by the stalk-reactive antibodies (Jegaskanda et al., 2017b), such correlates of protection cannot be used to evaluate the protective efficacy of a T cell epitope-based vaccine containing non-HA epitopes. Moreover, when using HA stalk-based vaccines, high levels of stalk-reactive antibodies represent a good protective efficacy against the same group IAVs (Figure 1). However, the binding affinities of HA stalk antibodies are variable among different viruses, and therefore may require different antibody titers to exhibit sufficient protection against the viruses. This speculation is supported by the observations that broadly reactive HA stalk antibodies show considerably different neutralizing abilities and binding affinities to the viruses within the same HA group (Ekiert et al., 2011, 2012). Furthermore, HA stalk-based vaccine and M2e-based vaccine may demonstrate a different balance of protective abilities to each other, between direct neutralization and indirect effector mechanisms (ADCC, for instance) by the respective antibodies. It has been shown that M2e antibodies provide protection via FcR-dependent effector functions rather than direct virus neutralization (Deng et al., 2015a), whereas HA stalk antibodies exert both direct neutralization and indirect effector functions (Krammer and Palese, 2015). The antibody-mediated inhibition of virus attachment measured by hemagglutinin inhibition assay or microneutralization assay is the gold standard for seasonal influenza vaccines (Ohmit et al., 2011). However, the assay cannot be applied simply to reflect the cross-protection by a UIV against heterologous/heterosubtypic influenza viruses. Therefore, it is essential to develop mechanistically distinct *in vitro* or *in vivo* assays to measure potential correlates of protection in order to evaluate the protection potency and breadth of the vaccine.

### Mode of Protection by a UIV

**Figure 4:**
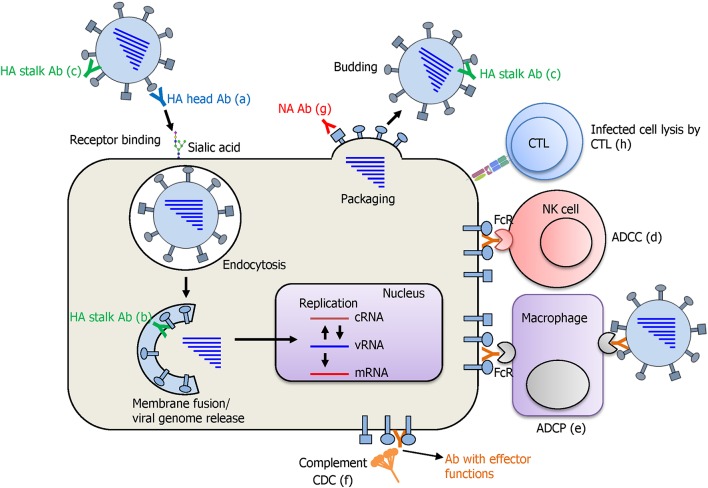
Protection mode of action afforded by a UIV. Antibodies against the HA globular head domain inhibit viral attachment via HA-mediated receptor binding to the sialic acid on cellular receptors (a). HA stalk antibodies have multiple protective functions. As the virus enters the cell, pre-bound stalk antibodies prevent the fusion of viral and endosomal membranes and block the viral genome release into cytoplasm of the cell (b). Binding of stalk antibodies can also limit the access of cellular proteases to the cleavage site located in the stalk domain and inhibit the cleavage and subsequent conformational change of HA that is an essential step for acquiring viral infectivity (c). Different antibodies against HA stalk and also other viral proteins such as NA, M2, and NP are shown to mediate antibody-dependent effector functions such as antibody-dependent cellular cytotoxicity (ADCC), antibody-dependent cellular phagocytosis (ADCP), and complement-dependent cytotoxicity (CDC), leading to the lysis of the virus-infected cells by immune cells or complement system (d–f). NA antibodies inhibit receptor destroying activity of NA and prevent the budding of newly formed viral particles from the cells (g). Cytotoxic T lymphocytes (CTLs) recognize the viral peptide presented on MHC-I molecule and kill the virus infected cell by the secretion of cytotoxic granules and cytokines (h).

The cornerstone of developing a UIV is the determination of the precise protection mechanisms of immune response against influenza viruses. Influenza HA recognizes sialic acid on the cellular receptors and initiates infection by entering the cell via receptor-mediated endocytosis (Figure 4). While HA inhibitory (HAI) antibodies have long been considered as the gold standard for strain-specific protection, very few of them were shown to elicit a broad protection by binding to the conserved receptor-binding site (RBS) of HA, thereby preventing viral entry to the cell (Krause et al., 2011; Ekiert et al., 2012). Recently, multifunctional protection mechanisms have been described for HA stalk-reactive antibodies. It has been shown that HA stalk antibodies may inhibit membrane fusion, the release of viral genome into the cytoplasm of the cell, and maturation of the HA precursor (Krammer and Palese, 2015). Furthermore, HA stalk antibodies can induce antibody-dependent effector functions such as antibody-dependent cellular cytotoxicity (ADCC), antibody-dependent cellular phagocytosis (ADCP), and complement-dependent cytolysis (CDC), resulting in clearance of virus-infected cells by the immune cells or the complement system (Jegaskanda et al., 2017b). During viral budding, NA cleaves the sialic acid from HA and supports multiple infection cycles by release of the newly assembled viral particles. NA inhibitory (NAI) antibodies specific to the conserved regions have shown an exceptional breadth, inhibiting divergent influenza viruses (Chen et al., 2018). In addition to the broadly protective antibodies, T cell immunity against conserved viral internal proteins also provides a broad protection. Cross-reactive cytotoxic T lymphocytes (CTLs) recognize the viral epitopes presented on MHC molecules and kill the infected cells. It is noteworthy that the cross-reactivity of T cell immunity has been recently shown to cover both IAVs and IBVs, and even the ICVs (Koutsakos et al., 2019), although its protective role *in vivo* has not been confirmed.

## Current Status of M2e-Based UIV Approaches

IAVs have two major surface proteins, HA and NA, and one minor surface protein, the M2 ion channel. During the infection cycle, the M2 ion channel is responsible for acidification of the viral interior, facilitating virus uncoating, and unloading of viral ribonucleoproteins (RNPs) into the host cytoplasm (Pinto et al., 1992). The extracellular domain of M2 protein (M2e) consists of 24 amino acids, among which 9 N-terminal amino acids are completely conserved among all IAVs (H1–H18) and minor mutations are observed in the distal portion (Deng et al., 2015a; Tsybalova et al., 2018). Therefore, M2e is considered as a promising target for eliciting broadly reactive antibodies. However, due to poor immunogenicity of the small M2e region, UIV approaches targeting M2e required carriers, vectors, and adjuvants to enhance immune responses (Deng et al., 2015a; Lee et al., 2015). One of the most efficient approaches is to make virus-like particles (VLPs) which display M2e on their surface. It has been shown that the hepatitis B virus core (HBc) protein fused with M2e self-assembled into VLPs that resemble wild type virus particles, expressing M2e on the surface (Neirynck et al., 1999). Following this work, substantial efforts were made to improve the immunogenicity and protective efficacy of M2e-HBc VLP vaccine constructs. Major strategies include the use of multiple copies of M2e by tandem repeats (Ravin et al., 2015; Sun et al., 2015; Tsybalova et al., 2015) and combination with adjuvants such as cholera toxin A1 (De Filette et al., 2006) or B subunit of *Escherichia coli* heat labile enterotoxin (LTB) (Zhang et al., 2009). The M2e-HBc VLP was further harnessed with T cell immunity by combining conserved T cell epitope from NP (Gao et al., 2013). The arginine-rich domain of HBc was shown to induce Th1-biased immune response of M2e-HBc VLP by binding to RNA, leading to improved protection (Ibanez et al., 2013). Besides the HBc, M2e was fused with various coat or capsid proteins derived from other viruses including Malva mosaic virus (Leclerc et al., 2013), tobacco mosaic virus (Petukhova et al., 2013), Papaya mosaic virus (Denis et al., 2008), T7 bacteriophage (Hashemi et al., 2012), and RNA phage Qβ (Bessa et al., 2008). Moreover, the enveloped VLPs displayed M2e in a membrane-anchored manner by combining influenza matrix protein and transmembrane (TM) domain of HA fused into M2e. The Sf9 insect cells infected with baculoviral vectors expressing influenza M1 and M2e-TM of influenza HA produced influenza VLPs displaying much higher levels of M2e than the wild type virions (Kim et al., 2015, 2017). Other vaccine types, including DNA vaccines or recombinant protein vaccines expressing M2e with various carriers to enhance immune responses, have also been advanced (Deng et al., 2015a).

Many studies conducted on animals and humans have elucidated the mechanisms of cross-protection conferred by M2e-based vaccines. A common observation is that antibodies specific to M2e cannot directly neutralize the viruses but confer cross-protection by eliciting several mechanisms of antibody-mediated and cell-mediated immune responses. The most well-characterized protection mechanisms include ADCC, ADCP, and CDC (Figure 4). Studies have shown that multiple types of immune cells including the natural killer (NK) cells, neutrophils, dendritic cells, or macrophages have the ability to sense the virus-infected cells by interaction with the Fc receptors (FcR) and the Fc region of M2e antibodies. This results in killing (ADCC) or phagocytosis (ADCP) of the infected cells (Huber et al., 2001; Jegerlehner et al., 2004; Hashimoto et al., 2007; Guilliams et al., 2014). The complement is also known to bind to the Fc of M2e antibodies and triggers the complement cascade, leading to formation of the membrane attack complex and target cell lysis (CDC) (Wang et al., 2008; Kim et al., 2018b). These studies together suggest that M2e-specific non-neutralizing antibodies play a crucial role in broad protection against IAVs through the clearance of the virus-infected cells. Besides non-neutralizing effector functions, it has also been shown that M2e-displaying recombinant bacteriophages induce HAI antibodies in a mouse model (Deng et al., 2015b). In addition, the T cell responses directed to M2e have been shown to contribute to cross-protection. Several studies have shown the presence of CD4 and CD8 T cell epitopes in M2e region in mice and humans (Jameson et al., 1998; Gianfrani et al., 2000; Mozdzanowska et al., 2003; Eliasson et al., 2008). In line with this, it has been demonstrated that CD4 T cell and CD8 T cell-mediated immunity are critical for cross-protection elicited by M2e-based UIVs including VLPs or recombinant peptide vaccines (Lee et al., 2014; Zhang et al., 2016; Arevalo et al., 2017; Pendzialek et al., 2017; Eliasson et al., 2018).

It remains a challenge to overcome the low protective efficacy of M2e-based vaccines, due to intrinsic low immunogenicity of the vaccine antigen and the low abundance of M2 proteins on influenza virions and infected cells. From the clinical and practical standpoints, multiple immunizations with a high dose of vaccine antigens combined with potent adjuvants may pose safety concerns, rendering M2e-based vaccine approaches inadequate as a stand-alone UIV. Consequently, M2e antigen was combined with other influenza viral antigens (mostly HA) or supplemented with strain-specific vaccines, resulting in improved potency, and broader cross-protection. For instance, several studies combined M2e with influenza HA head or stalk domain to elicit broadly protective immunity against heterologous influenza infections with various vaccine platforms including nanoparticles, recombinant proteins, and recombinant influenza viruses (Guo et al., 2017; Bernasconi et al., 2018; Deng et al., 2018; Tsybalova et al., 2018). Supplemented vaccination with M2e was examined to overcome the strain-specific protection or poor immunogenicity of classical inactivated or split vaccines (Music et al., 2016; Song et al., 2016). Besides the approaches described above, a vast amount of research on M2e-based UIVs have accumulated in the last decade, which are described in specialized reviews (Deng et al., 2015a).

## HA Stalk-Based UIV Approaches

### General Principles of HA Stalk-Based Approaches

**Figure 5:**
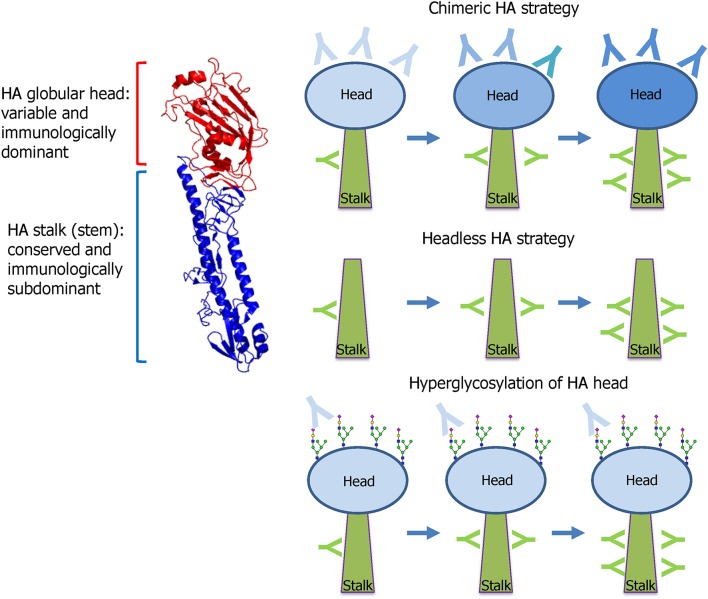
HA stalk-based approaches. Influenza HA comprises two functionally distinct domains; globular head and stalk. The globular head is variable among the viruses and immunologically dominant and, thus, the majority of neutralizing antibodies are directed to the head that harbors the receptor-binding site (RBS). The stalk is conserved and, therefore, stalk antibodies show broad protection against diverse strains within a group. However, the stalk is immunologically subdominant because of the physical shielding by the bulky head and the close proximity to the viral membrane. To preferably induce the broadly protective stalk-reactive antibodies, two vaccine approaches were designed; chimeric HA and headless HA. Chimeric HA consists of stalk domain derived from important target strains such as H1, H3, or B viruses fused with head domain from irrelevant avian strains such as H5, H6, or H8. Multiple immunizations with different chimeric HAs selectively increase stalk antibodies. Headless HA (stalk-only HA) lacking the head domain can induce only stalk antibodies through multiple vaccinations. Hyperglycosylation in antigenic sites in head domain results in the redirection of antibody responses toward stalk domain. The HA structure was downloaded from the Protein Data Bank (HA of A/swine/Iowa/15/1930, PDB ID 1RUY) and the final image was produced by PyMOL program).

The HA stalk-based approach currently leads the mainstream of UIV development. Influenza HA is the primary target antigen of the currently licensed seasonal vaccine, and HAI antibodies serve as the “gold standard” when evaluating the protective efficacy of HA-based vaccines (Ohmit et al., 2011). However, HA-based vaccines provide only strain-specific protection to antigenically homologous viruses, necessitating annual update of the surface antigens of seasonal vaccines to match the circulating viral strains. HA comprises two distinct domains; globular head, and stalk domains. The head domain is highly variable and immunologically dominant and thus the majority of HI antibodies are directed to the head domain that harbors the RBS (Figure 5). In contrast, the HA stalk domain is relatively conserved among the viruses, and therefore eliciting antibodies specific to the stalk is considered as a key to developing a UIV. However, the stalk is generally regarded as immunologically subdominant, due to masking effect by bulky head domain and close proximity to the viral membrane (Krammer and Palese, 2013; Crowe, 2018; Krammer, 2019), which necessitates rational design of a HA antigen and novel vaccination strategies to increase stalk-reactive antibodies. The major protective function of HA stalk antibodies is to lock the HA trimer in a prefusion state. This prevents pH-dependent conformational change of HA that triggers the membrane fusion and release of viral genome into cytoplasm of host cell (Ekiert et al., 2009). The membrane fusion inhibitory activity of the stalk antibodies may lead to neutralization of influenza viruses, although the neutralizing potency is weaker than head antibodies that directly prevent the receptor binding. Besides the neutralizing activity, stalk antibodies have multiple indirect mechanisms that contribute to broad protection, including antibody-dependent effector mechanisms (Jegaskanda et al., 2017b) and the inhibition of the NA enzymatic activity (Wohlbold et al., 2016; Chen et al., 2019). To overcome the low immunogenicity of HA stalk, genetic modifications were required for efficient exposure of stalk to the host immune system. Prime-boost vaccination with chimeric HAs or “headless” HAs were designed to boost stalk-reactive antibodies (Figure 5). Alternatively, hyperglycosylation in HA head resulted in the redirection of immune response from head to stalk (Krammer and Palese, 2015) (Figure 5). These approaches have paved the way to increase the breadth of protection against influenza viruses.

### Protection Mechanisms of HA Stalk-Based Vaccine

A primary function of stalk-reactive antibodies is membrane fusion inhibition. Following the influenza virus entry into the cells via endocytosis, a pH-dependent conformational change of HA proteins triggers the membrane fusion between the virus and endosome, leading to release of the viral genome into the cytoplasm. Binding of the antibody to the stalk interrupts the conformational change of HA and prevents subsequent membrane fusion. This leads to the trapping of the virus in the endosome, eventually aborting the infection (Ekiert et al., 2009; Sui et al., 2009). Moreover, binding of the stalk antibody blocks the access of proteases to the cleavage site located in stalk region and prevents protease-dependent cleavage of the HA precursor into HA1 and HA2 subunits, which is a prerequisite for the conformational change of HA (Ekiert et al., 2009; Brandenburg et al., 2013). These two overlapping functions of stalk-reactive antibodies result in inhibition of membrane fusion and direct neutralization of the virus. In addition to these mechanisms that operate at the early stage of infection, the stalk antibody may also bind to the newly expressed HA proteins on the cell surface, and interfere with the viral budding or release, at the later stage of infection (Tan et al., 2014).

In addition to direct neutralization, stalk antibodies have indirect mechanisms that involve diverse innate immune cells to clear the virus-infected cells from the host. The NK cells sense virus-infected cells via interaction between FcR and Fc portion of antibody bound to HA stalk expressed on the cell surface, and kill the infected cells by releasing cytotoxic granules and antiviral cytokines, via ADCC mechanism (DiLillo et al., 2014) (Figure 4). The FcR-Fc interaction is also required for ADCP, by which macrophages or neutrophils recognize and engulf antibody-bound influenza viral particles or infected cells (Ana-Sosa-Batiz et al., 2016). In addition, stalk antibodies bound to virus-infected cells are also able to activate the complement system, leading to the lysis of virus-infected cells (CDC) (Terajima et al., 2011). Animal challenge models have demonstrated broad protection by passive immunization with HA stalk monoclonal antibodies (DiLillo et al., 2014, 2016). The stalk-reactive antibodies also inhibit NA enzymatic activity through steric hindrance, limiting NA access to the HA-bound sialic acid, thereby preventing viral egress (Wohlbold et al., 2016; Chen et al., 2019; Kosik et al., 2019). The failure to release newly assembled virions from the infected cells prevents the viruses from entering into multiple cycles of infection. Overall, the stalk antibodies confer a broad protection from influenza virus infection through multiple mechanisms affecting both HA and NA throughout the infection cycle of influenza virus.

### HA Stalk-Based Vaccine Constructs

The first reported HA stalk-based UIV construct is the headless HA, a deletion mutant of HA lacking the globular head region (Graves et al., 1983; Sagawa et al., 1996; Steel et al., 2010) (Figure 5). While the initial strategies demonstrated broadly protective potentials in animal models, the removal of the head region often led to misfolding of the stalk immunogen, rendering the critical neutralizing epitopes ineffective. Subsequent studies were conducted to enhance the structural integrity of the stalk immunogens by rational designs to mimic the native trimeric conformation (Lu et al., 2014; Mallajosyula et al., 2014; Impagliazzo et al., 2015). Other studies designed and evaluated nanoparticle structures consisting of the stalk antigens for broad protection in mice and ferret models (Yassine et al., 2015; Corbett et al., 2019). The second approach to preferably induce stalk-reactive antibodies is based on chimeric HA that consists of stalk domain derived from the major target strains such as H1, H3, or B viruses fused with head domain from irrelevant avian strains such as H5, H6, or H8 (Krammer and Palese, 2015) (Figure 5). Multiple immunization was required to increase the stalk antibodies sufficient to provide protection from viruses within the same HA group (Hai et al., 2012; Krammer et al., 2013, 2014). The chimeric HA approach involves a full-length functional HA protein and, therefore, it may be expressed in genetically engineered viruses of the wild type or live attenuated influenza viruses (Krammer et al., 2013; Isakova-Sivak et al., 2018). Third approach relies on hyperglycosylation in the HA head domain (Figure 5). The introduction of additional N-glycosylation sites into the immunodominant head domain shields the major antigenic sites in the head and redirects the host immune responses toward the immune-subdominant stalk. The hyperglycosylated HA antigens induced higher stalk antibodies and provided better protection than the wild type HA in a mouse model (Eggink et al., 2014). Similar approaches were tested for avian H5N1 vaccines, in which hyperglycosylated HA antigens were delivered in various vaccine formats such as DNA, recombinant protein, VLP, and adenoviral vector (Lin et al., 2012, 2014). A recent study has shown that glycan shielding of HA head resulted in immune focus on a conserved epitope occluded at the head interface, with Fc-dependent protection activity in mice (Bajic et al., 2019a). These results together suggest that hyperglycosylation in the HA head is a promising strategy to find novel target epitopes hiding in the head and stalk in HA.

### Current Issues in HA Stalk-Based UIVs

#### Vaccine Efficacy

As discussed above, the protective efficacy of HA-stalk based vaccines is relatively weak, and multiple boost immunizations are required for efficient protection (Krammer et al., 2013, 2014). In addition, the breadth of protection is within the same HA group rather than both the groups (Krammer et al., 2013). To complement the low efficacy, prime-boost approaches that entail sequential immunization by LAIV followed by inactivated virus containing chimeric HAs are being evaluated. As examined by a ferret challenge model, this approach provided potent cross protection (Nachbagauer et al., 2017). It was observed that there is a “disconnect” between stalk antibody titers in serum and the protection level (Nachbagauer et al., 2017). The results show that the broad protection observed in the ferret model was primarily mediated by multiple-level immune responses by LAIV and rather than the stalk antibodies (see section Live attenuated influenza vaccine as an alternative strategy below). This observation in preclinical model leads to the question of the relevance of HA stalk antibodies to cross-protection in humans. Studies with human infection model have led to a somewhat different interpretation on the protective role of the stalk antibodies, depending on the study design. A human challenge study showed that the naturally occurring stalk antibody titers were predictive of lowering viral shedding, but demonstrated poor correlation with the reduction of clinical symptoms upon the pandemic A/H1N1 challenge (Park et al., 2018). In contrast, a more recent study suggested the preexisting HA stalk antibodies as an independent correlate of protection against natural infection with the pandemic A/H1N1 virus (Ng et al., 2019). Thus, further studies are required for a conclusive demonstration of the breadth and efficacy of cross-protection offered by HA stalk antibodies in human challenge models.

#### Potential Vaccine Safety Issues

Although the HA stalk based strategies have shown promising broad protection in animal models, potential safety issues have been raised (Crowe, 2018; Khurana, 2018). It is suggested that the stalk antibodies are responsible for antibody-dependent enhancement of viral infectivity in a swine model. In a study, pigs vaccinated with inactivated H1N2 vaccine developed more severe respiratory diseases upon heterologous H1N1 challenge as compared to the non-vaccinated control, which was ascribed to infectivity-enhancing effects of stalk antibodies (Khurana et al., 2013). Similarly, the HA subunit vaccine resulted in vaccine-associated enhanced respiratory disease (VAERD) in pigs after heterologous challenge (Gauger et al., 2011; Rajao et al., 2014). This effect cannot be generalized as the enhancement was noticed only in the adjuvanted antigen in a swine model, but not seen in similar studies with non-adjuvanted vaccines in a ferret model (Krammer et al., 2014). In addition, it has been recently shown that head-reactive, non-neutralizing monoclonal antibodies increase the stalk flexibility and membrane fusion kinetics, resulting in enhanced respiratory disease in a mouse model (Winarski et al., 2019). These results indicate that head-reactive antibodies may also induce antibody-dependent enhancement. However, similar observations in humans are debatable. For instance, humans immunized with 2008–2009 inactivated TIV exhibited an increase in illness following infection with 2009 H1N1 pandemic virus (Janjua et al., 2010; Skowronski et al., 2010) due to the presence of cross-reactive antibodies (Monsalvo et al., 2011). Similar fatal cases were observed during 1957 H2N2 pandemic, although the relation of HA stalk antibodies to these observations is not known. It is noteworthy that NAI antibodies could reduce VAERD caused by mismatched heterologous HA, suggesting that vaccines which target HA protein alone may be prone to VAERD and cross-protective NAI antibodies may counteract VAERD (Rajao et al., 2016). Although there is no report on VAERD associated with the headless or chimeric HA vaccines, careful monitoring is advised as these approaches rely heavily on stalk antibodies. The inclusion of NA antigens into HA stalk-based vaccine merits further evaluation. Another issue raised by a recent study is the auto-reactivity of stalk antibodies to human proteins, which is significantly higher than the head antibodies, as confirmed by multiple *in vitro* assays (Bajic et al., 2019b). Although biological implications of this observation have not been studied *in vivo*, it was speculated that vaccine strategies focused exclusively on the stalk, or any single conserved epitope may fail to induce adequate antibody titers due to negative selection of the auto-reactive B cell clones (Bajic et al., 2019b). However, clinical observations showed that, despite under potential negative selection, preexisting stalk antibodies conferred protection against the 2009 pandemic A/H1N1 infection in humans (Ng et al., 2019).

#### Stalk Variability

Although conserved among the influenza viruses, the HA stalk is not antigenically identical, showing sequence variability even within the same HA subtype. In line with this, a study demonstrated that H1 HA stalk could undergo mutations *in vitro* by immune pressure, although the variability was less than the head region (Anderson et al., 2017). On the other hand, another study show that while the stalk domain does evolve over time, this evolution is slow and, historically, is not directed to aid in evading neutralizing antibody responses (Kirkpatrick et al., 2018). Several studies have isolated mutant influenza viruses showing resistance to stalk antibodies (Ekiert et al., 2011; Friesen et al., 2014; Chai et al., 2016). Notably, all isolated resistant viruses were seen to harbor three different mutations (Gln387Lys, Asp391Tyr, and Asp391Gly) in stalk region. While Gln387Lys mutation completely abolished the binding of the antibody to stalk region, the other two mutations rarely affected the antibody binding but enhanced the fusion ability of HA, representing two independent resistance mechanisms of the virus to escape stalk-reactive antibodies (Chai et al., 2016). These reports together show that the stalk may undergo natural or directed antigenic changes, asking important considerations in the context of developing stalk-based UIVs. Especially, the occurrence of mutations that enhance the membrane fusion of HA in presence of stalk antibodies presents a potential concern to vaccine safety issues. It should, however, be noted that the stalk antibody escape mutants tend to lose a viral fitness and are highly attenuated *in vivo* (Henry Dunand et al., 2015; Chai et al., 2016), alleviating the safety concerns.

#### Rational Design of Stalk

It is well-recognized that the stalk is less immunogenic than head and that the stalk antibodies are less potent in virus neutralization than the head antibodies. Therefore, multiple immunizations (three or four times) with stalk-based vaccines are required for inducing a sufficiently protective level of antibody response. More importantly, most humans have a diverse history of exposure to influenza antigens, by natural infection, or vaccination. The established immune memory influences the subsequent immune response to a UIV, posing a great challenge to rendering qualitatively uniform and protective immune responses in humans. As discussed earlier, the HA stalk-based vaccines elicit broad-spectrum protection within the same HA group, but usually fail to provide protection to strains belonging to different HA group (Krammer et al., 2013; Margine et al., 2013). These results apparently dissent from the finding that some of the stalk monoclonal antibodies isolated from humans recognize all IAV subtypes, neutralizing both group 1 and group 2 viruses, presenting a promising prospect of developing pan-influenza A therapeutic solution (Corti et al., 2011). Isolation of rare antibodies with extremely broad neutralization potency from humans with prior vaccinations or infections (Corti et al., 2011) indicates that our immune system is able to find the cryptic and conserved epitopes and generate specific antibodies to the regions. However, inducing such antibodies to a protective level by vaccination remains a big challenge. Some recent reports encourage other options such as the activation of the conserved “cryptic” epitopes via antigen processing mechanisms (Lee et al., 2018b) or deliberately down-regulating surface antigens (Yang et al., 2013). Moreover, Fc-engineering technologies developed to enhance the therapeutic efficacy of antibodies may be harnessed to modulate FcR-Fc mediated effector functions (DiLillo et al., 2014) to achieve broad protection. A comprehensive understanding of immune response to broadly neutralizing epitopes and structure-based antigen design is required for the rational design of a pan-influenza A vaccine covering both group 1 and group 2 IAVs, as a consensus criterion of UIV (Table 1).

## Conserved Targets in HA Other Than Stalk

The inability to induce a complete protection by HA stalk-based approach led to a search for alternative targets for UIVs. Human monoclonal antibody CH65, mimicking the interaction with sialic acid, was shown to bind to the RBS in HA and neutralize multiple H1N1 influenza strains (Whittle et al., 2011). A caveat is that this antibody demonstrated stringent structural requirements for neutralization and mutation at the binding region led to generation of escape mutants. A panel of head-reactive monoclonal antibodies was also isolated and shown to recognize conserved region in the RBS and neutralize multiple influenza viruses (Krause et al., 2011, 2012; Ekiert et al., 2012; Benjamin et al., 2014).

The HA head domain was also reported to contain conserved epitopes outside the RBS. Different broadly neutralizing antibodies recognized the conserved regions located in HA head domain and neutralized multiple influenza viruses without detectable HI activity (Iba et al., 2014; Raymond et al., 2018). Generally, neutralization breadth of head antibodies was considerably variable to each other, ranging from pan-subtype (covering the same subtype) to pan-type (covering both group 1 and 2) coverage, indicating the presence of variably conserved regions. Additionally, a novel class of cross-reactive antibodies was discovered in humans vaccinated with seasonal TIV (Lee et al., 2016). These antibodies were shown to bind to a highly conserved region located on the HA RBS that was occluded in the HA trimer, conferring protective immunity against H1N1 and H3N2 strains *in vivo*, without neutralizing activity *in vitro*. Furthermore, a novel class of antibodies targeting vestigial esterase (VE) domain in HA has been characterized. The VE domain consists of two non-continuous sequences in HA head domain, which together forms a structurally distinct subdomain from the RBS and HA stalk domain (Zheng et al., 2018). The VE domain of the HEF protein of ICV is responsible for cleaving the host receptor to facilitate viral release, whereas in IAVs and IBVs the same receptor cleaving function is provided by a separate NA. The VE domains are found in both IAVs and IBVs, although their functions are not well-defined. The VE domains are highly conserved within a subtype of IAV but are variable among different subtypes (Ha et al., 2002). Monoclonal antibodies to the VE domain of H5N1 virus demonstrated broad neutralization against multiple clades of H5N1 subtype by preventing viral entry into cells (Oh et al., 2010). To date, different monoclonal antibodies have been isolated and they bind to different epitopes in the VE domain of H5N1 viruses, suggesting the presence of multiple neutralization epitopes in the VE domain (Paul et al., 2017). The VE-binding antibodies are reported to mediate ADCC for *in vivo* protection via FcR-Fc interaction (Wang et al., 2017). Besides H5N1, several monoclonal antibodies neutralizing H3 or H7 of IAVs or IBVs have also been recently characterized (Tan et al., 2016; Chai et al., 2017; Bangaru et al., 2018). Taken together, possibility remains to identify conserved neutralizing epitopes in the head domain in HA, in addition to extensively characterized HA stalk. Activation of these epitopes via antigen processing machineries (Lee et al., 2018b) may offer an option for enhancing the potency of cross-protection.

Despite the presence of conserved epitopes, the head domain is not a feasible vaccine antigen because of the immunodominance of the surrounding variable regions in the head that compete and prevent effective induction of antibodies toward the conserved regions. Therefore, several strategies were designed to enhance the breadth of protection by HA-based vaccines. The centralized HA was reconstructed such that whole HA contained consensus amino acids derived from diverse strains within a subtype (Weaver et al., 2011). This engineered HA antigen induced stronger immune response and provided better protection against heterologous influenza viruses, as compared to natural wild type HA antigen. Another approach, conceptually similar to the centralized HA, is based on a computationally optimized broadly reactive antigen (COBRA), in which the HA was designed to carry consensus sequences. The COBRA strategy was tested against H1N1, H3N2, and H5N1 influenza viruses, and demonstrated broad protection within a subtype (Giles and Ross, 2011; Crevar et al., 2015; Carter et al., 2016). A third strategy is the use of ancestral sequences as vaccine antigens to widen the window of cross-protection against diversified lineages or clades. Through phylogenetic tree analysis, putative ancestral HA and NA sequences have been determined and used as vaccine antigens, showing broadened cross-protection against multiple clades of H5N1 viruses in animal models (Ducatez et al., 2011, 2013). Collectively, the UIV candidates using HA head or full-length antigen are based on reconstructed HA containing consensus sequences. Although the protection breadth of those strategies appears to be restricted to a subtype or a specific clade, similar concepts may be applied to other antigens such as HA stalk or NA to substantially improve the protection breadth.

## NA as a Novel Target For a UIV

### Multiple Function of NA in Infection Cycle

NA is a tetrameric type II transmembrane glycoprotein and the second major surface protein of influenza viruses. The role of NA in influenza infection cycle is classically known as an expedited release of virus particle from infected cells by cleaving off the sialic acid residue present in host cell membrane, thus enabling multiple rounds of infection by the newly generated viral progeny. In addition to the canonical role that operates at later stage of infection, other functions of NA relevant to the infection cycle are being recognized. For instance, the sialidase activity of NA is critical for viral entry into a host cell at early stage of infection. At the entry site in the mucus, influenza virus meets mucosal defense proteins such as mucins that are highly glycosylated and capture viral HA. NA is able to release the captured viral particles via sialidase activity, allowing them to reach the host cells successfully (Cohen et al., 2013; Yang et al., 2014). Furthermore, with the same sialidase activity, NA facilitates HA-dependent membrane fusion and enhances the viral infectivity by removing the sialic acid residues from the virion-expressed HAs (Su et al., 2010). Additionally, NA cooperates with HA to enable crawling and gliding motions of influenza virus on cell surface to enhance viral entry into a cell (Sakai et al., 2017). More interestingly, some of the H3N2 viruses use their NA for receptor binding instead of HA, suggesting a novel role of NA other than receptor-destroying activity (Lin et al., 2010; Mogling et al., 2017). These observations show that NA performs multiple functions in the entire infection cycle, suggesting that NA antibodies may represent an important means of protection against influenza viruses.

### NA Antibodies as Important Correlate of Protection

The 1968 H3N2 pandemic gave us important lessons pertaining to NA-mediated protection. The antigenic drift of NA is independent of HA; the pandemic involved a shift in HA, but NA remained close to the previous influenza A/H2N2 viruses (Schulman and Kilbourne, 1969). Notably, it has been shown that individuals with higher N2 antibody titers are less likely to be infected with the H3N2 pandemic, depicting the contribution of NA antibodies to broad protection (Schulman, 1969; Murphy et al., 1972; Monto and Kendal, 1973). However, NA has been largely ignored in the formulation of influenza vaccines due to the general beliefs that NA antibodies do not inhibit viral entry and that NA is less abundant than HA on a virion. Furthermore, the lack of a convenient assay to measure functional NA antibodies has rendered the NA forgotten antigens in influenza vaccines for decades (Eichelberger and Monto, 2019). Most of the current vaccine approaches focus on the induction of HA antibodies, both in trivalent/quadrivalent seasonal influenza vaccines and in the recent UIV candidates. However, it has been increasingly acknowledged that NA antibodies are important and independent correlates of protection and that NA immunity should be considered when evaluating vaccine potency. Clinical studies have shown a correlation between vaccination-induced or preexisting NAI antibody levels and decreased frequency of influenza infection and illness (Couch et al., 2013; Monto et al., 2015; Park et al., 2018). Further, a human challenge model depicted that NAI titers correlated more significantly with protection and disease severity than HAI titers (Memoli et al., 2016), or even HA stalk antibodies (Park et al., 2018). The observations in the human challenge models suggest that NA should be given full consideration as a vaccine antigen for better protection.

Several animal studies have identified NAI monoclonal antibodies that show protective effects against heterologous influenza infection. The breadth of NAI antibodies varied from subtype-specific to pan-influenza, depending on the conserved epitopes (Doyle et al., 2013a,b; Wan et al., 2013). Recently, it was reported that influenza infection in humans induces a variety of broadly reactive antibodies directed to the NA (Chen et al., 2018). In this study, it was shown that among the total influenza-specific antibodies induced by infection, the NA-reactive antibodies accounted for 23% and HA-reactive antibodies 35%. By contrast, the subunit or split vaccine resulted in antibody response directed predominantly to HA (87%), with only 1% for NA. The poor ability of the seasonal vaccine to induce NA antibodies was apparently due to insufficient content or structural integrity of NA antigen used in current vaccine formulation. This research suggests that correctly folded and immunologically relevant NA antigen is capable of inducing broadly protective antibody responses.

### NA-Based Vaccine as Low-Hanging Fruit for a UIV?

Although the importance of NA-immunity in protection against homologous and heterologous influenza infections is clearly established, only a few literatures have demonstrated the cross-protection of NA-only vaccine constructs. One recent study in a mouse model has reported that computationally engineered recombinant NA proteins containing consensus sequences show broad protection within the H1N1 subtype (Job et al., 2018). Some other studies reported that VLPs expressing NA provided cross-protection against heterologous challenge in mice and ferrets (Easterbrook et al., 2012; Walz et al., 2018; Kim et al., 2019), and recombinant NA in a mouse model (Liu et al., 2015; Wohlbold et al., 2015). However, co-administration of H7 HA and N3 NA in modified vaccinia virus Ankara (MVA) vectors did not demonstrate enhanced protection efficacy as compared to the efficacy of MVA-HA or MVA-NA vaccine alone (Meseda et al., 2018). A predominant immune response in favor of HA over NA, when presented in an influenza virion, is already well-documented (Johansson et al., 1987), and the dissociation of HA and NA eliminates this antigenic competition (Johansson and Kilbourne, 1993, 1996). These observations together suggest that NA-specific immunity may be dwarfed by competition with highly immunogenic HA in the final vaccine formulation. It could be argued that if the controlled ratio of HA and NA (dwarfing NA) is the strategy adopted by successful virus infection to minimize the host immune surveillance, then a deliberate perturbation of their ratio (increasing NA) in the vaccine formulation may be a promising strategy for effective protection. It was shown that the ratio of HA/NA could vary widely (up to 50 fold) without affecting viral fitness by a single mutation in the viral promoter (Lee and Seong, 1998). It remains to be seen if such a reverse-genetic approach could be harnessed to enhance the potency of NA-based vaccines.

Currently, we have very limited knowledge about anti-NA immunity. To develop a broadly protective vaccine based on NA, there are several critical questions that need to be answered. Firstly, although the NAI antibodies have been determined as an independent correlate of protection in humans (Couch et al., 2013; Monto et al., 2015), this needs to be further validated by the NA-only vaccine constructs in animal and human models. Secondly, very little is known about the breadth of NA immunity. The literature discussed earlier has consistently demonstrated a subtype-specific protection (e.g., within N1 or N2) of NA-based vaccines in animal models. Considering the repertoire of influenza viruses infecting humans and animals (including N1, N2, N3, N7, and N9 encompassing both NA group 1 and 2) (Figure 1), a successful NA-based vaccine should be designed to elicit broad protection covering both NA groups. Hence, the determination of conserved regions or epitopes hidden in NA is urgently needed. Thirdly, the mechanisms by which NA antibodies contribute to protection are not completely understood. Many NAI antibodies inhibit its enzymatic activity and thus prevent the release of newly formed viral particles. However, the extent to which NAI antibody titers may be considered protective has not been determined yet. Evaluation of cross-protection against mismatched or heterologous strains may be even more complicated. While ADCC was shown to be involved in protection by non-neutralizing NA antibodies (Jegaskanda et al., 2014, 2017a; Wohlbold et al., 2017), other protective mechanisms are yet to be further elucidated. Further isolation and characterization of broadly protective NA antibodies is required for better design of NA-based vaccines. Comprehensive reviews on NA-based immunity and the perspectives on current knowledge gaps to be addressed may be found in specialized reviews (Wohlbold and Krammer, 2014; Krammer et al., 2018a).

## UIV Against Influenza B Viruses

Besides IAVs, ~25% of all human influenza virus infections in each epidemic season is caused by IBVs that are classified into two distinct lineages, Victoria-like and Yamagata-like lineages (Ambrose and Levin, 2012). The current seasonal influenza vaccine is prepared in a trivalent or quadrivalent formulation, depending on the inclusion of one or two lineages of IBV antigens. Although priority is given to IAVs owing to a greater impact, IBVs may be more vulnerable targets against which to develop a UIV (Table 1) because of their low antigenic diversity and lack of animal reservoir (Figure 1) (Tan et al., 2018). Indirect evidence is being accumulated by the isolation of cross-protective antibodies against IBVs. Several broadly protective antibodies binding to head or stalk domain of influenza B HA have been isolated in humans. Overall, these monoclonal antibodies show lineage-specific neutralizing activity *in vitro*. Further, *in vivo* protection against both lineages was also demonstrated in mice by passive transfer, through non-neutralizing antibody-dependent effector functions (Shen et al., 2017; Hirano et al., 2018; Vigil et al., 2018; Asthagiri Arunkumar et al., 2019; Liu et al., 2019b). Notably, some of B HA stalk antibodies demonstrated extremely broad binding ability (Hirano et al., 2018) or protection against both IAVs and IBVs (Dreyfus et al., 2012). Influenza B NA-reactive broadly neutralizing antibodies were also isolated in animals and humans. Seasonal QIV induced NA antibodies with broad and potent antiviral activity against both lineages in humans (Piepenbrink et al., 2019). Additionally, murine NA antibodies also showed broad protection against both lineages of IBV (Wohlbold et al., 2017).

In line with these observations, chimeric HA strategy has also been tested for a UIV against IBVs. Chimeric HAs consisting of HA head domain from IAV and stalk domain from IBV, delivered as a DNA vaccine (prime), followed by two boosting immunizations with protein vaccines into mice, provided broad protection against both the lineages as well as an ancestral strain of IBV (Ermler et al., 2017). Mosaic HA is an advanced version, in which major antigenic sites of type B HA head domain were replaced by those of type A HA head so that antibodies directed to conserved regions both in the head and stalk domains of type B HA could be induced (Sun et al., 2019). The mosaic B HA provided broad protection against both lineages of IBV, through non-neutralizing ADCC-mediating antibody responses. There are only a few studies reporting B NA-based vaccine offering cross-lineage protection. A study showed that mice immunized with recombinant B NA protein of B/Yamagata/16/88 were protected from homologous Yamagata-like and Victoria-like lineages (Wohlbold et al., 2015). Another study demonstrated that a B NA-based vaccine inhibited the transmission of both homologous and heterologous IBVs in Guinea pig model (McMahon et al., 2019). As compared to IAVs, very little effort has been made so far to develop a UIV against IBVs. However, considering the limited diversity and variability (Figure 1) compared to IAVs, further identifications of broadly protective B cell and T cell epitopes would make it possible to develop a pan-influenza B vaccine in the near future (Tan et al., 2018).

## T cell Immunity as an Essential Factor for Truly Universal Influenza Protection

A vast majority of current efforts to develop a UIV are focused on inducing antibody response toward surface glycoproteins, M2e, HA, and NA. However, T cell immunity has been acknowledged as a potential immune correlate of broad protection against influenza infections (Sridhar, 2016; Clemens et al., 2018). T cell immunity may not provide sterilizing or neutralizing immunity against influenza viruses but improves the standard of care by reducing the disease severity and duration of infection, facilitating recovery from illness (Sridhar, 2016). It, therefore, seems that multiple immune arms including both antibodies and T cell immunity are critical to provide a truly universal protection against highly variable influenza viruses. The influenza-specific T cell immunity is known to be highly cross-reactive by recognition of conserved peptides between different subtypes of IAV (Assarsson et al., 2008; Kreijtz et al., 2008; Lee et al., 2008; van de Sandt et al., 2014). Extensive studies have proven the protective role of vaccination or infection-induced cross-reactive CD8+ T cells in various animal models (Kreijtz et al., 2007, 2009; Bodewes et al., 2011; Hillaire et al., 2011). Additionally, in humans, CD8+ T cells offered cross-protection across seasonal, pandemic, avian IAVs, and both lineages of IBVs (Gras et al., 2010; Hayward et al., 2015; van de Sandt et al., 2015; Wang et al., 2018b). The majority of cross-reactive T cell epitopes of IAVs are derived from internal proteins; among >70 T cell epitopes identified between H5N1 and H3N2 viruses, only one derived from HA and other from internal proteins (Lee et al., 2008). This is not surprising given that the conservation rate of internal proteins is >90%, whereas that of surface HA and NA is only 40–70% among IAVs (Lee et al., 2008), which shows that inducing T cell immunity directed to internal proteins of influenza virus may provide a basis of developing T cell-based UIVs.

Despite poor sequence homology between the HAs of IAVs and IBVs, HA stalk harbors not only extremely broad B cell epitopes but also T cell epitopes encompassing both types of influenza viruses. The fusion peptide in HA stalk contains a cross-reactive CD4+ T cell epitope conserved in both IAVs and IBVs, although its protective role has not been examined *in vivo* (Babon et al., 2012). A number of CD4+ and CD8+ T cell epitopes are highly conserved in internal proteins (Terajima et al., 2013). A recent study has discovered a universal human CD8+ T cell epitope in PB1 (NMLSTVLGV PB1_413−412_) that is identical across IAVs, IBVs, and ICVs (Koutsakos et al., 2019). The preexisting memory PB1_413−412_+CD8+ T cells were detected in the blood and lung tissues of healthy donors and clonally expanded upon infection with IAV or IBV. This report not only showed the existence of heterotypic memory CD8+ T cells in humans that could be induced by exposure to influenza viruses, but also presents the prospect of designing a T cell-based UIV. However, these cross-reactive T cells were not induced in HHD-A2 mouse model despite multiple influenza infections or vaccinations and the protective role of the T cells could not be assessed in the study. Nonetheless, the existence of a number of cross-reactive T cell epitopes between IAVs and IBVs provides an avenue to address to a UIV.

Several T cell-based vaccine candidates are in different stages of clinical development, the major underlying strategy of which is to deliver multiple T cell epitopes derived from different viral antigens including internal as well as surface antigens (Sridhar, 2016). Delivery platforms include replicating or non-replicating viral vectors derived from vaccinia or adenovirus, recombinant VLPs, recombinant protein or peptide vaccines, and DNA vaccines (Sridhar, 2016; Clemens et al., 2018). Modified vaccinia Ankara (MVA) vector encoding NP and M1 was shown to induce robust T cell responses and provide cross-protection against multiple subtypes in animals and humans (Antrobus et al., 2012; Powell et al., 2013; Hessel et al., 2014; Folegatti et al., 2019). The baculovirus VLPs carrying influenza HA/NA and M1 offered cross-protection where T cells played a significant role in protection in mice (Hemann et al., 2013; Keshavarz et al., 2019). Synthetic peptides or fusion proteins harboring multiple conserved T cell epitopes have also been evaluated for immunogenicity and protective efficacy in animal models (Adar et al., 2009; Rosendahl Huber et al., 2015). While the vaccine approaches described above deliver exogenous antigens and induce CD4+ T cells as well as CD8+ T cells by cross-presentation, DNA vaccines are designed to predominantly activate cytotoxic CD8+ T cells to recognize endogenously expressed antigens presented on MHC class I molecules. In fact, the first report on DNA vaccines was targeted to influenza virus (Cohen, 1993; Ulmer et al., 1993), but initial success in a mouse model did not well-translate into higher animal models due to poor performance (Porter and Raviprakash, 2017). To date, much progress has been made to improve the efficacy of DNA vaccine against influenza virus, encompassing rational design of antigens and expression vectors and the development of novel adjuvants and delivery methods (Lee et al., 2018a). Candidate universal DNA vaccines encoding NP, matrix proteins, or PB1 were shown to decrease viral load and cross-protect against heterologous challenges in diverse animal models including mice, pigs, ferrets, and macaques (Ulmer et al., 1993; Tompkins et al., 2007; Price et al., 2009; Bragstad et al., 2013; Wang et al., 2015; Koday et al., 2017). Further studies are required for to refine DNA vaccine approaches as a stand-alone UIV. Recent studies have indicated that DNA vaccines may serve an attractive component of prime-boost strategy, considering it as a very effective means to priming immune system when preexisting immunity is low (Ledgerwood et al., 2013; DeZure et al., 2017). Despite the potential for broad protection, the safety issues need to be monitored closely, especially because of the documented rise in pathological consequences associated with CTL responses (Peiris et al., 2010; Duan and Thomas, 2016).

## Live Attenuated Influenza Vaccine as an Alternative Strategy

### Cross-Protective Immunogenicity of LAIV

LAIV has been approved for clinical use in humans since 2003 and is proven effective in preventing influenza infections (Mohn et al., 2018). The protection of LAIV is superior to IIVs due to multifaceted immune arms including antibody responses and cell-mediated responses that operate systemically and locally (Jang and Seong, 2013a,b; Sridhar et al., 2015). Further, displaying a whole set of viral antigens in a native conformation into the host immune system presents a definite advantage of LAIV to generate better protective immunity than the other strategies relying on a limited set of antigens. As discussed above, T cell immunity directed to the conserved viral epitopes constitutes the cornerstone of cross-protection. A large number of researches have shown that T cell responses induced by LAIV are critical for broad protection against heterologous and heterosubtypic influenza infections in animal models (Cheng et al., 2013; Jang and Seong, 2013a; Rekstin et al., 2017). In young children, only LAIVs were shown to induce durable and potent T cell responses, while developing similar levels of antibody response as compared to IIVs (Belshe et al., 2000; Hoft et al., 2011; Mohn et al., 2015, 2017). Despite well-documented cross-protection, LAIV has received little attention to develop a UIV. This may be attributed to the general belief that LAIV is not effective at inducing broadly neutralizing antibodies against conserved domains in surface antigens (e.g., M2e or HA stalk). However, close attention is recently being given to LAIV as an alternative platform as a potent and cross-protective vehicle than previously thought, through inducing multifaceted immune correlates including T cell response and antibody-mediated effector functions (Jang and Seong, 2013a, 2014).

### UIV Approaches Using LAIV

In the UIV approaches reported so far, LAIV was used either as a component in prime-boost regimens with other different vaccine formats such as IIV, DNA vaccine, or recombinant protein vaccine. Alternatively, LAIVs were also studies as a stand-alone vaccine given in single or multiple doses. A reassortant LAIV expressing a chimeric HA was constructed under the genetic background of Russian strain (A/Leningrad/134/17/57 cold-adapted virus) and used as a boosting vaccine in a ferret model (Nachbagauer et al., 2017). Notably, the LAIV-IIV regimen showed greater protective efficacy against the pandemic H1N1 challenge than the IIV-IIV regimen in terms of viral loads in the respiratory tissues, despite 32-fold lower stalk antibody titers in serum. Several factors were presumed to account for this disconnect between stalk antibody titers and protection efficacy, including anti-NA immunity, mucosal IgA antibodies, cell-mediated immunity, and non-specific innate immune responses offered by the LAIV. Similar results were obtained when using a different LAIV strain (A/Ann Arbor/6/60 cold-adapted strain) (Nachbagauer et al., 2018). In a subsequent study performed by the same group, chimeric HA-containing the LAIV-LAIV (A/Leningrad/134/17/57 cold-adapted strain) vaccine regimen was compared with the LAIV-IIV combination in terms of protection efficacy in a ferret model, in which the LAIV-LAIV vaccine regimen conferred superior protection against pandemic H1N1 and H6N1 challenges than the LAIV-IIV (Liu et al., 2019a). Another group tested a vaccination regimen comprising only LAIV (A/Leningrad/134/17/57 cold-adapted strain) as prime-boost vaccination in a mouse model (Isakova-Sivak et al., 2018). To enhance the breadth of protection, an internal gene of cold-adapted virus was replaced with the wild type. This study compared the immunogenicity and cross-protection between chimeric HA-containing LAIVs and natural HA-containing LAIVs. The chimeric HA-containing LAIVs induced higher HA stalk antibody titers and showed better cross-protection against heterologous infection with various group 1 IAVs. Thus, a cooperative role of cell-mediated immunity and HA stalk antibodies was suggested, although their individual contribution to protection were not assessed directly. It would be interesting to investigate if the cross-protection could be extended to group 2 influenza viruses such as H3 or H7 strains.

Besides the Leningrad and Ann Arbor strains, an independent cold-adapted vaccine strain (X-31ca) that was previously used for trivalent seasonal vaccine (Jang et al., 2014), H5N1 pre-pandemic vaccine (Jang et al., 2013c), and 2009 pdmH1N1 vaccine (Jang et al., 2012, 2013a), was recently tested as a UIV in a mouse model (Jang et al., 2018). Mice vaccinated with single or two doses of X-31 ca LAIVs were completely protected against lethal challenge of heterosubtypic strains encompassing both HA group 1 and 2 IAVs. Interestingly, boosting with heterosubtypic LAIVs carrying different HA and NA surface antigens showed more potent cross-protection than homologous boosting. T cell immunity and NK cell-mediated ADCC activity was demonstrated to contribute significantly to the observed cross-protection. As the first report of pan-influenza A protection covering both HA groups, these results merit further studies in a ferret model for clinical relevance. Hence, the LAIVs appear to be a powerful tool to develop a UIV that confers broad and potent cross-protection as a stand-alone vaccine or in combination with other strategies.

### Future Prospects of LAIV-Based UIVs

While the LAIV presents a remarkable prospect for a broadly protective influenza vaccine, several important issues need to be addressed for it to serve as a reliable vaccine modality. The protection efficacy of a LAIV substantially differs with age. The estimated protection efficacy of a LAIV is 80% in young children and only 40% in adults, to the matched strains (Jefferson et al., 2008, 2010). As for T cell immune responses, clinical studies indicate that LAIVs induce better T cell response than IIVs in both children and adults (He et al., 2006; Subbramanian et al., 2010; Hoft et al., 2011). However, clinical studies have reported that LAIVs are not effective at inducing T cell responses in adults and the elderly, perhaps due to preexisting “vector” immunity, which limits the replication of LAIVs (He et al., 2006; Forrest et al., 2008; Hoft et al., 2011). Considering that both humoral and cell-mediated immunity contribute to broad protection, it will be important to elucidate how preexisting immunity or immunologic imprinting affects B cell and T cell immune response induced by LAIVs in humans (Gostic et al., 2016; Henry et al., 2018). For this purpose, animal models mimicking preexisting immunity and controlled human challenge studies will be needed. Considering that LAIVs mimic natural infection, a fundamental question remains to be answered: if infection (or vaccination with LAIV) is effective for conferring protection, why are humans vulnerable to repeated infections with homologous or heterologous strains? At the population level, currently used cold-adapted LAIVs provide a relatively low protection rates (~40% in adults) even against matched strains (Jefferson et al., 2010). However, little is known whether individuals who successfully acquired protective immunity by a cold-adapted LAIV were protected against other heterologous strain(s) in the next epidemic. This may be directly addressed by a well-controlled longitudinal cohort study using human challenge models. Some studies showed that LAIVs were able to generate cross-reactive T cell responses in children for up to 1 year after vaccination, as a basis of long-term cross-protection in humans (Mohn et al., 2015, 2017).

Most of the LAIV-based approaches are based on cold-adapted attenuated strains (Nachbagauer et al., 2017, 2018; Jang et al., 2018; Liu et al., 2019a). These strains acquire multiple attenuation mutations in the internal genes during the cold-adaptation process, which contribute to genetic stability to overall attenuation (Jang et al., 2016). A common concern for live vaccines is safety issues, especially those associated with potential reversion of attenuating mutation(s) into virulence during vaccination. LAIVs acquired multiple attenuation mutations among various internal genes and proven safe as seasonal influenza vaccines (especially for A/Ann Arbor strain as the master strain for FluMist). However, genetically engineered LAIVs with a limited set of attenuation mutations, e.g., NS1-deletion or elastase-dependent HA cleavage (Talon et al., 2000; Stech et al., 2005), may require additional monitoring on safety.

Defining the precise correlates of protection represents the most challenging step in the development of a UIV (Erbelding et al., 2018). Significant efforts were made to identify the protection mechanisms of HA stalk-based vaccines, suggesting that direct neutralization in combination with Fc-dependent indirect effector mechanisms mediated by stalk antibodies were the primary correlates of protection. In contrast to HA stalk-based vaccines, LAIVs elicit multiple immunological factors including serum IgG antibodies and mucosal IgA antibodies to surface antigens (HA, NA, and M2) and cell-mediated immunity to internal antigens, synergistically contributing to protection. However, their quantitative relationship to protection has not been determined, not even for homologous protection (Sridhar et al., 2015; Mohn et al., 2018), let alone for cross-protection against heterologous infection. The development of standardized assays to quantitatively measure T cell-mediated protection is particularly challenging, as the magnitude and the subset of T cells critical for protection is likely to differ according to strains of LAIV and challenge viruses. Further, mucosal IgA antibodies are believed to correlate with cross-protection, but it is still challenging to measure the neutralizing activity or effector functions of mucosal IgA antibodies. The complicated nature of LAIV-induced immunity, including non-neutralizing antibodies and diverse subsets of T cells, present a bottleneck to identifying precise correlates of protection.

**Figure 6:**
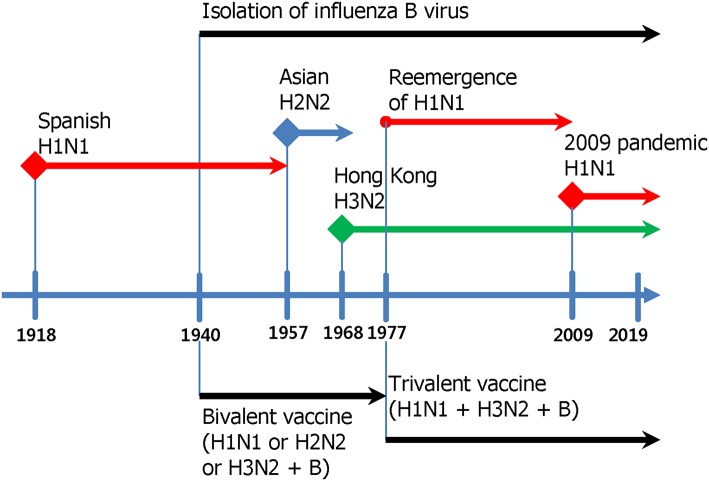
Co-evolution of influenza viruses and influenza vaccines. Within the past century, there were four influenza pandemics; 1918 Spanish flu (H1N1), 1957 Asian flu (H2N2), 1968 Hong Kong flu (H3N2), and 2009 swine flu (H1N1) (Saunders-Hastings and Krewski, 2016). The 1918 Spanish flu (H1N1) evolved into seasonal influenza strain and had circulated for ~40 years until the next pandemic by 1957 Asian flu (H2N2), which after ~10 years of circulation was replaced by 1968 Hong Kong flu (H3N2). The 1968 Hong Kong flu (H3N2) has circulated until now as seasonal influenza strains. In 1977, H1N1 strain reemerged and was replaced by the 2009 swine flu (H1N1), which evolved into seasonal influenza strains circulating until now. Thus, H1N1 and H3N2 strains began to co-circulate from 1977. After the reemergence of H1N1 in 1977, World Health Organization has issued recommendations for trivalent vaccine composition containing A/H1N1, A/H3N2, and B strains (Hannoun, 2013).

Another important aspect of LAIV-based strategies lies on the LAIV strains. During the past century, H1N1 and H3N2 subtypes of influenza A viruses were the most prevalent strains in humans, causing annual epidemics as well as occasional pandemics, except for the temporal circulation of H2N2 during 1957–1968 (Kilbourne, 2006) (Figure 6). Accordingly, seasonal influenza vaccines are recommended to include H1N1 and H3N2 vaccine antigens for more than 40 years since 1977. Therefore, it is likely that most contemporary population has preexisting T cell immunity to H1N1 and H3N2 strains through natural infections or vaccinations. It should be remembered that currently licensed LAIVs (A/Ann Arbor/6/60 ca and A/Leningrad/134/17/57 ca) are of H2N2 subtypes. Probably, the nature of strain itself does not really matter for seasonal influenza vaccine, for which strain-specific immunity is focused on the surface HA antigen. However, for eliciting cross-protection, the role of conserved region become important (see section HA stalk-based UIV approaches, Conserved targets in HA other than stalk, NA as a novel target for a UIV for HA/NA and section T cell immunity as an essential factor for truly universal influenza protection for internal proteins). It is likely that human populations under 50 years of age (born after 1968 when H2N2 became extinct) has little preexisting immunity against H2N2, but predominantly against H1N1 and H3N2 viruses. It will therefore be worthwhile to examine whether cold-adapted LAIVs of H1N1 (Jang et al., 2018) or H3N2 origin (non-existent, to our knowledge) offer a beneficial effects on boosting the preexisting cross-reactive T cell immunity and antibody effector functions (section Mode of protection by a UIV; Figure 3).

As for IBVs, two distinct lineages diverged and circulated after 1985, which necessitates the incorporation of Victoria-like and Yamagata-like lineages in seasonal influenza vaccines. This leads us to raise a possibility of using an ancestral influenza B strain (such as B/Lee/40) before divergence into two different lineages as a UIV candidate for IBVs. Establishing new cold-adapted LAIV strains is a time-consuming and laborious. However, recent advances in reverse genetics and rational approaches to attenuate viral virulence have enabled the rapid conversion of a wild type virus into a novel LAIV strain. These approaches include NS1 truncation, elastase-dependent HA cleavage, caspase-dependent NP and NS1 cleavage, microRNA-mediated silencing, and codon deoptimization (Talon et al., 2000; Stech et al., 2005; Coleman et al., 2008; Perez et al., 2009; Jang et al., 2013b).

The redirection of host immune responses from surface proteins toward internal proteins may be achieved by rational vaccination strategies with LAIVs. For example, the down regulation of expression levels of HA or NA in a LAIV (Yang et al., 2013) is likely to result in preferable induction of T cell immunity to internal proteins. Alternatively, vaccination with LAIV carrying HA from non-human influenza viruses such as H5 or H9 may be effective at boosting preexisting T cell immunity to internal proteins in humans. Given the cross-reactivity of T cell immunity between IAVs and IBVs, A type LAIVs and B type LAIVs may be administered as a bivalent formulation or by sequential vaccination to induce improved protection.

## Other Strategies for UIVs

A number of alternative strategies are being investigated for their potential to serve as a UIV platform. First approach is to use infection-competent but replication-defective viruses. For instance, the M2 knock-out influenza virus (M2SR) rescued from M2-producing cells is able to infect cells but does not produce progeny virus due to the lack of functional M2 protein. The M2SR was shown to induce strong cell-mediated and humoral immunity and to provide broad protection against both homologous and heterologous influenza virus challenge in mice and ferrets (Sarawar et al., 2016; Hatta et al., 2017, 2018). A conceptually similar to M2SR, PB2 knock-out influenza virus could be produced in PB2-expressing cells as a vaccine. The protection efficacy of PB2-KO vaccine was tested against diverse influenza strains in mice (Victor et al., 2012; Uraki et al., 2013; Ui et al., 2017). Pseudotyped influenza A virus also presents a similar replication-incompetent virus approach. The pseudotyped influenza virus lacking HA produced in HA-expressing cells can infect cells and express all the viral proteins except for HA. Studies have shown that the vaccination of mice and ferrets with the pseudotyped influenza virus generated a vigorous T cell response and reduced lung viral loads and weight loss after challenge with homologous and heterologous influenza viruses (Powell et al., 2012; Baz et al., 2015). These approaches based on replication-defective viruses should be rigorously evaluated for safety, considering potential reversion into virulence by acquiring the wild type gene by reassortment with circulating viruses (Lowen, 2017).

Secondly, mRNA vaccines have emerged as a promising alternative to conventional vaccine platforms against various infectious diseases including the influenza virus (Pardi et al., 2018a). Initially, the mRNA vaccines successfully induced protective B cell and T cell immune response in mice, ferrets, and pigs (Petsch et al., 2012). In subsequent studies, mRNA vaccines encoding HA induced neutralizing antibodies and T cell immunity essential for heterologous protection in mice and ferrets (Brazzoli et al., 2016). Notably, mRNA vaccines encoding HA elicited HA stalk antibodies in mice, rabbits, and ferrets, and the stalk antibody responses were associated with protection against homologous and heterologous influenza virus infection in mice (Pardi et al., 2018b), depicting promising potential as a UIV platform.

## Conclusions and Prospects

During the past few years, several critically important issues have emerged in developing a UIV. While identification of a number of broadly protective antibodies presents an optimistic prospect for pan-influenza therapeutics, induction of such antibodies at a sufficiently protective level by vaccination has not been accomplished yet. A variety of viral targets have been identified to induce broad protection, including M2e and the conserved regions in the HA, such as stalk, RBS, and VE, and the long-neglected NA has emerged as an essential target for durable and broad protection. In virtually all cases, the rationale for broad protection is to redirect the immune responses from the variable immunodominant regions to conserved immunosubdominant regions. While effective in eliciting cross-protection, concerns were raised to non-neutralizing antibodies that potentially trigger the enhancement of disease. T-cell immunity has also been considered as an important correlate of cross-protection. LAIVs are able to induce multifaceted immune response and thus have increasingly received close attention as a promising vaccine platform, either alone, or in boosting format. Preexisting immunity or immunologic imprinting, established by prior exposure to influenza by infection or vaccination, may influence or thwart the desired breadth of immune response by UIVs. Therefore, disparities may exist between pre-clinical evaluation of a UIV without due recognition of immune imprinting and human challenge studies. Lastly, the correlates of protection and their precise molecular mechanisms have not been determined yet, which remains a significant gap between development and licensing of a UIV. Given the limited antigenic diversity of IBVs among humans and the lack of animal reservoir, pan-influenza B virus vaccine may be attainable in the near future. On the other hand, IAVs continually change their antigenicity via antigenic drift and shift and zoonotic spillover from animal reservoirs, requiring multi-faceted immune arms to increase the breadth of protection. Judicious choice of antigens and their efficient designing to confer a broad protection for a prolonged period are required to counterfeit the immune evasion and provide a truly universal protection against the ever-changing influenza viruses.

## Author Contributions

BS designed and conceived the review. YJ and BS investigated the literature and wrote the manuscript.

### Conflict of Interest

The authors declare that the research was conducted in the absence of any commercial or financial relationships that could be construed as a potential conflict of interest.
